# 射频消融治疗100例肺部肿瘤的远期疗效

**DOI:** 10.3779/j.issn.1009-3419.2011.04.06

**Published:** 2011-04-20

**Authors:** 宝东 刘, 磊 刘, 岩 李, 鸿 王, 牧 胡, 坤 钱, 若天 王, 修益 支

**Affiliations:** 1 100053 北京，首都医科大学宣武医院胸外科 Department of Toracic Surgery, Xuanwu Hospital, Capital Medical University, Beijing 100053, China; 2 100053 北京，首都医科大学宣武医院放射科 Department of Radiology, Xuanwu Hospital, Capital Medical University, Beijing 100053, China

**Keywords:** 肺肿瘤, 射频消融, 生存, Lung neoplasms, Radiofrequency ablation, Survival

## Abstract

**背景与目的:**

手术切除是治疗肺部肿瘤的首选治疗手段，而对高龄、肺功能差的患者，射频消融治疗是一个很好的选择。本文总结了射频消融治疗100例肺部肿瘤的远期疗效和并发症。

**方法:**

对不能手术的100例肺部肿瘤患者行射频消融治疗，定期复查胸部增强CT扫描、肿瘤SPECT或PET显像并随访，观察远期疗效及并发症。

**结果:**

100例肺部肿瘤病例的106个病灶接受了射频消融治疗。其中男性62例，女性38例，年龄36岁-91岁，平均66.6岁。原发性肺癌86例，肺转移瘤14例。所有病例完成射频消融治疗，无严重并发症和围手术期死亡。全组总生存时间为13.0个月，1年生存率51.0%，2年生存率32.5%，原发性肺癌与肺转移瘤相比无统计学差异（*P*=0.922）。早期肺癌的中位生存时间为28个月，2年总生存率为57.7%。

**结论:**

CT引导下射频消融治疗肺癌具有安全、有效、定位准确和微创的优点，是不能耐受手术的早期肺癌患者的选择之一，对中晚期肺癌是一种局部减瘤手段。

## Abstract (en)

**Background and objective:**

Surgical resection is the preferred treatment in selected patients with pulmonary neoplasms. In older than 70 years or have compromised cardiopulmonary status or coexistent medical problems patients, radiofrequency ablation (RFA) may offer an alternative option. The aim of this study is to evaluate the therapeutic effects afer RFA in 100 patients with pulmonary neoplasms.

**Methods:**

One handred cases of unresectable lung tumors with 106 lesions were underwent RFA therapy. To evaluate the therapeutic effect and complications of lung tumors using spiral CT scanning and SPECT in 1-3 months afer RFA.

**Results:**

One hundred patients underwent RFA for lung neoplasms (62 men, 38 women; median age, 66.6 years; range, 36 to 91 years). Eighty-six patients with primary lung neoplasms and 14 patients with pulmonary metastases underwent RFA. Treatment was complete in all cases, no treatment-related deaths occurred in all of the 100 patients and serious morbidity associated with the procedures. The median overall survival for the entire group of patients was 13.0 months, the one and two years overall survival for total of were 51% and 32.5% respectively. No differences in overall survival noted between patients with primary and metastases lung neoplasms (*P*=0.922). The median overall survival for the early stage of patients was 28.0 months, 2-year overall survival for early stage primary lung cancer patients were 57.7%.

**Conclusion:**

RFA is a safe and effective procedure in selective lung tumors. CT-guided radiofrequency ablation is a minimally invasive treatment option. RFA could act as an alternative treatment to inoperable lung cancer.

肺癌由于缺乏有效的早期诊断方法，80%以上的病人就诊时已属晚期，并且部分患者因身体或年龄等因素不能耐受手术治疗，失去了手术切除的最佳时机。近年来，射频消融术（radio-frequency ablation, RFA）作为一种新的局部治疗手段，具有微创、安全、可靠、可重复进行等优点。我院自2006年开始将RFA用于肺部肿瘤的治疗，取得较好的近期疗效^[1]^。本文回顾性总结了100例肺部肿瘤病例射频消融治疗的远期结果，报告如下。

## 1 材料与方法

### 1.1 临床资料

2006年10月-2009年2月我院100例肺部肿瘤病人的106个病灶接受了射频消融治疗，其中男性62例，女性38例，年龄36岁-91岁，平均66.6岁。右上肺28例、右中肺3例、右下肺27例、左上肺27例、左下肺13例、双肺2例。患者术前诊断为肺癌或肺转移瘤，或者手术中获取病理学诊断（开胸肿物活检或者电视胸腔镜下胸膜活检、经皮肺穿刺活检），包括腺癌（包括细支气管肺泡癌）49例、鳞癌28例、腺鳞癌1例、小细胞癌5例、大细胞癌3例，肺转移瘤14例（包括胸膜间皮瘤3例、乳癌2例、结直肠癌2例、泌尿系移行上皮癌2例、食管癌或肉瘤2例、肺平滑肌肉瘤2例、耳腺样囊性癌1例）。肺癌分期：Ⅰa期2例、Ⅰb期5例、Ⅱb期5例、Ⅲa期9例（T3N1 5例、T1N2 4例）、Ⅲb期16例（T4N2 12例、T2N3 4例）、Ⅳ期49例（包括骨转移、脑转移、肝转移、对侧肺内转移）。术前常规检查包括肿瘤标志物、T/B淋巴细胞亚群、胸部CT、肿瘤SPECT或PET、腹部B超、骨扫描、头颅核磁等。

### 1.2 射频消融技术

使用RITA产品（RITA射频针StarBurst XL，StarBurst Xli，RITA射频发生器）。CT引导下射频消融治疗时考虑到患者存在自主呼吸，肺活动度较大，因此选择锚状射频针以便出针后固定肿瘤，减少射频针对肺的副损伤。采用64排CT扫描，根据CT扫描三维重建结果，确定穿刺点、深度和角度。局部麻醉（2%利多卡因）后，避开肋骨、大血管、肺大疱，将射频针按事先测得的方向和角度快速到达病变部位。射频针进入的深度以病灶内边缘为宜，然后再进行扫描，观察针尖若为最佳位置，推下射频针尾端使锚状电极从鞘内针尖端呈“伞”状弹出，再次扫描观察电极在病灶中的位置，如位置不理想，收回锚状电极，调整位置，重新弹出锚状电极，根据病灶大小设定出针长度，保证消融灶边缘超过肿瘤边缘0.5 cm-1 cm，以杀死肿瘤生长最活跃的周边部分。射频针尾部连接射频发生器，开始消融。一般消融靶温度设定在90 ℃，消融时间根据病灶大小设定，多点温度监测。直径 < 5 cm的肿瘤单次治疗即可，而对于直径 > 5 cm的病灶，最好根据CT增强和三维重建技术，准确显示肿瘤大小和形状以及附近关系，采用多针穿刺多层面适形消融或使用7 cm的针（StarBurst Xli, RITA）进行消融。射频消融治疗程序如表 1。

**1:** 肺部肿瘤射频消融治疗程序 Radiofrequency ablation protocol according to tumour size in lung neoplasms

Tumor diameter	Time at target temperature 90℃ at each dgree of electrode deployment^*^ (min)					
	2 cm	3 cm	4 cm	5 cm	6 cm	7 cm
≤ 1 cm	10	…	…	…	…	…
> 1 cm, ≤ 2 cm	#	15	…	…	…	…
> 2 cm, ≤ 3 cm	#	5	15	…	…	…
> 3 cm, ≤ 4 cm	#	2	5	20	…	…
> 4 cm, ≤ 5 cm	#	2	5	5	20	…
> 5 cm	#	2	5	5	5	20
^*^Maximum degree of electrode deployment was based on tumour size with the aim to induce a spherical volume of coagulation necrosis including target tumour and at least a 0.5 cm safety margin all around; #Time spent achieving target temperature of 90 ℃.						

开胸和电视胸腔镜射频消融按开胸术后常规处理。CT引导下射频消融完毕、冷却后收回锚状电极，针道消融，拔出射频针，包扎穿刺点，再次CT扫描，观察病灶有无变化和气胸出血等并发症，确定患者无异常时返回病房，静卧2 h。预防性使用抗菌素，发热、咳血等给予对症处理。根据患者病情给予放化疗和生物靶向治疗，同期处理其他转移灶，部分患者根据复查结果再次接受射频消融治疗。

### 1.3 随访与统计学处理

定期复查胸部增强CT扫描（肿瘤大小以射频消融后5 min扫描结果为基准）、肿瘤SPECT或PET显像（T/N值≥2.5判断为恶性）并随访。统计学处理采用SPSS 16.0软件，使用*Kaplan-Meier*生存分析、*Logrank*检验，以*P* < 0.05为有统计学差异。

## 2 结果

### 2.1 一般情况

100例肺部肿瘤患者接受射频消融治疗的病灶共106个，病灶直径最小1.0 cm，最大13.5 cm，平均4.2 cm。射频消融时间15 min-120 min，平均消融时间39.3 min。部分患者升温较慢，到达靶温度的时间一般超过4 min，考虑原因为腺癌的血管较鳞癌丰富而容易带走热量。开胸射频消融2例、电视胸腔镜下射频消融9例、CT引导下射频消融89例。

### 2.2 CT和SPECT显像改变

射频消融后5 min，CT扫描立即显示所有病例的病灶经治疗后阴影增大，体积平均增大约50%。病灶密度由原来的增强实质型转变成低密度，部分病灶出现液化坏死或空泡样改变，CT值下降者占97.2%（103/106），平均降至20以下，原因在于病灶在加热过程中瘤组织凝固性坏死、水肿、气化所致。病灶周边还同时出现磨砂玻璃样反应带，可能是由于加热后正常组织的急性炎性反应渗出所致。

术后1个月复查CT，病灶与原肿瘤相比变化不明显，而SPECT显示病灶T/N值下降至正常范围。术后3个月复查CT，与原病灶比较部分病灶消失或者缩小约0.5 cm-2 cm，并出现凝固性坏死区，SPECT显示病灶部分无核素浓聚。7例增大的病灶进行了第二次消融，其中6例在第一次消融后半年左右，1例在1年左右。

### 2.3 并发症

无射频消融治疗操作相关死亡。CT引导下射频消融术中一般感觉局部发热、出汗、甚至心率加快，无需特殊处理。术中咳血的5例患者经对症止血治疗未影响射频消融治疗；剧烈咳嗽6例，可能与刺激支气管有关，经过注水孔注入利多卡因缓解；13例出现气胸的患者经过抽气后仅1例回病房后再次出现大量气胸而行胸腔闭式引流术；皮下气肿1例；心率失常2例；局部疼痛6例，经过术中给予哌替啶或吗啡对症治疗好转；10例出现胸膜反应，降低靶温度到70 ℃，经过数分钟后部分患者靶温度可逐渐升至90 ℃。

### 2.4 随访

经随访，目前已有28例患者死亡，其中5例于术后1个月-2个月内死亡，2例于术后2个月-3个月之间死亡，6例于术后3个月-4个月内死亡，5例于术后4个月-6个月内死亡，8例于术后6个月-1年内死亡，1例于术后1年以上死亡。死因主要为中晚期肺癌远处转移或者严重合并症（慢性阻塞性肺疾病）。中位生存时间为13.0个月，1年生存率51.0%，2年生存率32.5%（图 1A）。原发性肺癌与转移性肺癌的生存率相比无统计学差异（*P*=0.922）（图 1B）。Ⅰ/Ⅱ期肺癌的中位生存时间为28个月，1年生存率82.5%，2年生存率57.7%（图 1C）。

**1:**
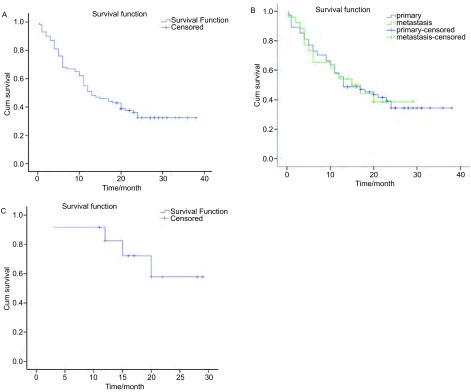
肺部肿瘤经过RFA治疗后的生存曲线。A：全部病例；B：原发性肺癌与肺转移瘤的比较；C：早期肺癌。 Survival curve of lung neoplasms after radiofrequency ablation. A: Total cases; B: Primary lung cancer and metastasis lung tumor; C: Early stage lung cancer.

## 3 讨论

Ⅰ/Ⅱ期肺癌按照美国国家癌症综合网络（National Comprehensive Cancer Network, NCCN）^[2]^和美国胸科医师协会（American College of Chest Physicians, ACCP）^[3]^的非小细胞肺癌（non-small cell lung cancer, NSCLC）临床指引：适合手术者应该进行解剖学肺叶切除加纵隔淋巴结清扫或采样，不适合肺叶切除者应该采用亚肺叶切除（首选肺段切除，次选楔形切除），不适合手术者应该选择射频消融或立体定向放射治疗。近10年来，肺叶切除作为早期肺癌的标准治疗模式已经受到挑战，在不久的将来，很难想象早期肺癌的局部控制会仅有一种治疗模式，患者可有局限性切除、立体定向放射治疗或射频消融等多种选择。一项回顾性研究^[4]^比较了不适合肺叶切除的亚肺叶切除（25例）和射频消融（22例）的治疗结果，研究结果显示总生存率和肿瘤特异性生存均无统计学差异。

射频消融是对靶肿瘤施以频率460 KHz-500 KHz的射频电流使肿瘤组织内的极性分子处于一种激励状态，发生高速震荡摩擦而产生热能。局部温度达到39 ℃-40 ℃可导致癌细胞停止分裂；41 ℃-42 ℃可杀死癌细胞；45 ℃-50 ℃细胞内的蛋白质变性、脂质层溶解、细胞膜破坏、组织细胞凝固性坏死；80 ℃-90 ℃可有效地快速杀死局部肿瘤细胞，同时肿瘤周围的血管组织凝固形成一个反应带，不能继续向肿瘤供血，有利于防止肿瘤转移。由于肺部正常组织可通过肺部大血管的血液循环和呼气散热，并且起着绝缘的效果，使能量可以充分集中在病变部位，加之肺部肿瘤组织的血流量低，散热困难，热量积聚，温度升高快，成为一个巨大的储热库，因此肺肿瘤非常适合射频消融治疗。由于射频消融能治疗肺肿瘤，又不损伤正常肺组织，因此为心肺功能差、不能耐受手术的肺癌患者提供了一种新的治疗方法。Dupuy等^[5]^2000年将这一技术用于3例肺癌的治疗。

射频消融治疗肺部肿瘤的主要适应症^[6]^：①高龄不能耐受手术的周围型早期肺癌患者；②因心肺功能差不能耐受手术的周围型肺癌患者；③不愿意接受手术的周围型早期肺癌患者；④肿瘤距离大血管或较大支气管在1.0 cm以上；⑤肺部转移瘤，数目 < 3个或直径总和 < 10 cm。国外文献报道射频消融治疗肺部肿瘤的适应证锁定在早期NSCLC和肺转移瘤，但鉴于国内的实际情况和伦理学情况愿意接受射频消融治疗的肺癌患者更多属于中晚期（不能手术）^[1]^，本组中76%（65/86）的肺癌病例属于晚期。

开胸射频消融的主要适应症是术中发现肿物不能切除，电视胸腔镜射频消融的主要适应症是术前发现有大量胸腔积液需要胸膜活检和胸膜固定。除以上情况外，我们均选择CT引导下射频消融。与开胸或电视胸腔镜下射频消融相比，CT引导下射频消融具有微创、及时发现并发症和易评价疗效等优点。

在系统性回顾研究中^[7]^，与操作有关的并发症发生率为15.2%-55.6%，死亡率为0-5.6%。最常见的并发症是气胸，发生率为4.5%-61.1%，大部分可以自愈，只有3.3%-38.9%（平均11%）需要放置胸腔闭式引流。本组无操作相关死亡，气胸发生率为13%（13/100），其中1例行胸腔闭式引流（7.69%）。本研究小组有丰富的肺穿刺活检经验，避免发生气胸的关键是穿刺技术要熟练，进针速度快和穿刺准确，需要调整射频针的位置时针尖尽量留在肺内或肿瘤内^[8]^。出现少量气胸不需特殊处理，中大量气胸需要抽净气体，防止影响射频针在肿瘤内的布针。

患者在治疗中发生咳嗽多数与射频针或热气刺激支气管有关，剧烈咳嗽者可给可待因止咳或注水孔注入利多卡因。血痰与穿刺损伤或者治疗后组织炎性反应有关，可予止血对症治疗。CT引导下射频消融治疗肺癌由于是在局麻下进行，部分贴近壁层胸膜的病灶在射频时刺激胸膜，出现疼痛或胸膜反应，经过降低靶温度到70 ℃，数分钟（3 min-5 min）后部分患者靶温度可升到90 ℃。术后70%的患者发热，一般为吸收热，热程在3 d-5 d，体温在38.5 ℃以下。肿瘤病灶较大者，发热温度较高，但一般不超过39 ℃。术后胸痛发生率约30%，术后48 h较重，一般止痛处理好转。

射频消融后1个月-3个月内病灶增大，因此1个月内CT评价疗效存在缺陷，此时病灶周围反应性充血、纤维组织增生一般还未消失，CT的变化难以与残留或复发作鉴别。3个月后病灶逐渐缩小，呈低密度改变。因此建议评估疗效以射频消融后5 min或者治疗后1个月的大小为基线进行评估，3个月以后评价疗效。FDG-PET比增强CT扫描判定疗效更为准确，至少也要3个月后评价疗效^[9]^。

一项系统性回顾研究^[7]^显示，射频消融治疗Ⅰ期NSCLC的局部复发率为3%-38.1%（平均11.2%），平均疾病无进展时间为15个月-26.7个月（平均21个月），1年、2年、3年生存率分别为63%-85%、55%-65%和15%-46%。近年研究提示射频消融治疗早期肺癌取得良好疗效。Hiraki等^[10]^对20例不能手术的Ⅰ期NSCLC进行射频消融治疗，1年、2年、3年的肿瘤局部控制率分别为72%、63%和63%；平均生存时间42个月。Simon等^[11]^对Ⅰ期NSCLC进行射频消融治疗，1年、2年、3年、4年、5年生存率分别为78%、57%、36%、27%和27%，5年无病生存率 < 3 cm者为47%，≥3 cm者为25%。Lanuti等^[12]^对31例不能手术的Ⅰ期NSCLC进行38例次射频消融治疗，经过4.5年的随访，局部肿瘤进展率31.5%，中位生存时间30个月，2年、3年生存率分别为78%、47%。Pennathur等^[13]^报道46例未分期的原发性肺癌射频消融治疗结果，2年生存率为50%（95%CI: 33%-65%）。Lencioni等^[6]^第一个发表了关于CT引导RFA治疗33例NSCLC的前瞻性多中心临床研究RAPTURE结果，1年、2年生存率分别为70%（95% CI: 51%-83%）和48%（95% CI: 30%-65%）；其中Ⅰ期NSCLC（*n*=13）的2年总生存率为75%（45%-92%），2年肿瘤特异性生存率为92%（66%-99%）。本组病例也得到类似的结果，因此有研究者^[14]^提出射频消融治疗早期肺癌可以代替肺叶切除。

对中晚期肺癌的射频消融治疗效果报道较少^[1]^。中央型肺癌肿块靠近肺门大血管、大气管，血流或空气带走大量热能，肿瘤内热量不易蓄积，难以形成凝固性坏死，因此疗效较差。一般认为，当肿瘤放疗时氧在放射破坏DNA并杀死肿瘤细胞方面是不可或缺的，因此放疗对肿瘤边缘的富氧细胞非常有效，但是放疗对肿瘤中心区的乏氧细胞效果较差，这部分肿瘤细胞通过加热（射频消融）可以杀死，因此两者具有互补作用，RFA联合放疗可以增加治疗效果。本组对中心型肺癌和右上肺纵隔型肺癌合并上腔静脉综合征的肺癌射频消融术后补充放疗，取得良好效果。因此，所有中晚期肺癌患者还需要全身治疗，包括化疗和靶向治疗等，可以提高疗效，但是射频消融在肺癌综合治疗中的作用还需进一步研究。

总之，CT引导下射频消融治疗肺癌具有安全、有效、定位准确和微创的优点，是不能耐受手术的早期肺癌患者的选择之一，对中晚期肺癌是一种局部减瘤手段。
